# Assessing the Genetic Diversity of Daylily Germplasm Using SSR Markers: Implications for Daylily Breeding

**DOI:** 10.3390/plants12091752

**Published:** 2023-04-25

**Authors:** Edvinas Misiukevičius, Birutė Frercks, Jūratė Bronė Šikšnianienė, Zygmunt Kącki, Małgorzata Gębala, Paulina Akulytė, Emilija Trilikauskaitė, Vidmantas Stanys

**Affiliations:** 1 Lithuanian Research Centre for Agriculture and Forestry, Institute of Horticulture, Department of Orchard Plant Genetics and Biotechnology, Kaunas District, LT-54333 Babtai, Lithuania; 2 Arboretum Wojsławice Botanical Garden, Faculty of Biological Sciences, University of Wroclaw, 58-230 Niemcza, Poland; 3 Institute of Biology Systems and Genetic Research, Lithuanian University of Health Sciences, LT-50161 Kaunas, Lithuania

**Keywords:** *Hemerocallis* spp., genetic diversity, microsatellite markers, breeding

## Abstract

This work aims to characterize the genetic diversity of species, early hybrids, and cultivars using microsatellite simple sequence repeat (SSR) markers, as well as analyze and identify the origin of *Hemerocallis* spp. early hybrids. For this research, samples were collected from different types of daylily species, early hybrids (known or hypothetically first-generation hybrids from *Hemerocallis* species), foreign, and Lithuanian varieties. An initial screening of SSR primers developed for *Hemerocallis citrina* was performed, and their suitability for testing other daylily species and hybrids was evaluated. The genetic diversity was assessed with the selected eight-primer set, and molecular SSR profiles were created. Primer SAU00097 is the most informative according to heterozygosity (0.95) and polymorphism information content (*PIC*) (0.17). The highest heterozygosity was observed in Lithuanian cultivars (0.713), the lowest in species (0.583). Genetic relationships between species show that only fulvous daylilies are separated into a different cluster. The highest variation among genotypes was observed in the species group (18%), while modern cultivars had the slightest variation among genotypes (1%). The putative origin of early hybrids was analyzed using a likelihood heatmap of all genotypes. Results show what species might be used in breeding for early hybrids. Several modern diploid and tetraploid daylily cultivars have triploid species as ancestors.

## 1. Introduction

Daylilies are one of the most popular ornamental plants worldwide. One flower’s lifespan lasts only from 9 to almost 17 hours [1], but the plant has many buds that continuously bloom for about a month. It is a herbaceous perennial plant that has been intensely hybridized for over a century. It shows significant economic value in the horticultural trade, and the large morphological diversity of hybrid cultivars makes this plant genus a future model plant for botanical and genetic studies [2]. Daylilies have been cultivated for thousands of years [3], mainly for flower bud consumption with exceptional medicinal value [4]. The daylily flowers have a lot of phenolic compounds [5], bioactive phenylpropanoids, and flavonoids for improving depression-like behavior [6]. The current study assumes that daylilies can be used as a functional food [5].

*Hemerocallis* was initially assigned to the *Liliaceae* family by Linnaeus in 1753. However, now it is attributed as belonging to the *Asphodelaceae* family [2]. Daylilies are naturally distributed in East Asia, with the paramount diversity of species originating in Korea, Japan, and China [7], with about 17 species known to genera. Daylily plants are found in grasslands and mountain habitats [8]. *H. citrina* is self-incompatible [9,10]; thus, hybrids of this species arise from an outcross and may represent distinctly different genotypes [11]. Daylily species introduced to the United States of America by Arlow Stout were used for breeding new varieties [12] that later were used in breeding programs. The daylily breeders, in most cases, use only phenotypical selection so that the collection could be falsely increased or decreased [13]. The molecular fingerprinting of cultivars would be helpful for the exact identification and authorization of new cultivars.

The base germplasm for new variety development in Europe was daylily cultivars from the U.S.A. and older germplasm collections in botanical gardens. European hybridizers do not have as deep of traditions in breeding daylilies as Americans. The European daylily breeding program was mostly limited in 2015 during the *Xyllela* spp. outbreak [14], as daylilies were considered hosts for invasive bacteria, and imports from third countries, including the U.S.A.—the primary source of new and more advanced cultivars—were forced to be limited. As climate conditions change, it is necessary to introduce new traits into European daylily breeding programs. One of the best ways to introduce new characteristics such as plant vigor, scape height, resistance, flower shape and diameter, and flower number and color is by using interspecific hybridization from wild species into cultivars [15]. There are two primary sources of genetic diversity in daylilies: wild populations and existing cultivars. Wild populations are essential because they represent the largest source of genetic variation for most traits [16]. To determine the uniqueness of the genotypes, the evaluation of morphological characteristics alone is insufficient, as they depend on different environmental conditions.

The most reliable way to identify the cultivars’ genetic diversity and origin is by using molecular markers [17,18,19]. The short sequence repeat (SSR) microsatellites are co-dominant markers suitable for pedigree studies [20,21,22] and breeding using marker-assisted selection [23,24,25]. SSR markers are even suitable on molecular evolutionary distant landrace studies [26,27]. Specific SSR markers were developed and used for *H. middendorffii* species characterization [28,29]. The universal SSR markers were developed for *H. citrina* and used for evaluating genetic diversity and population structure of daylily collections [30]. Although they have been developed for *H. citrina*, they were also determined to be suitable for other species and cultivars. Over the years, there has been confusion with daylily species classification [11,31,32]. Due to different environmental conditions and soil types, an artificial increase or decrease in the collections is possible, as plants that look phenotypically different can be genetically identical and vice versa [33]. The use of high-level molecular characterization techniques is needed for the secure conservation of genetic resources. The daylily genetic pool used in European breeding programs has not been evaluated with molecular markers.

Therefore, the aim of this work is to analyze and identify the origin of *Hemerocallis* spp. early hybrids, as well as characterize the genetic diversity of species, early hybrids, and cultivars using microsatellite (SSR) markers.

## 2. Results

### 2.1. SSR Primer Informativeness

To maximize the chances of producing new cultivars, the genetic value of each type of source material was evaluated by genotyping different daylily genotypes (Table S1). Ten microsatellite loci were used to evaluate the number of alleles, heterozygosity, and the polymorphism information content (PIC). Not all primers were informative. As with primers SAU00008 and SAU00048, no PCR products were generated. The highest number of alleles was generated with SAU00042, SAU00096, and SAU00097 SSR primers. The PIC value was moderate for all SSR primers, with an average of 0.15 (Table 1). The most informative primers were SAU00097 and SAU00006, with a PIC value of 0.17. According to the observed heterozygosity value, the primer SAU00097 is the most polymorphic (0.95). The least informative primers used in this study were SAU00042 and SAU00096, with a *PIC* value of 0.13.

The PIC values and heterozygosity of eight SSR primers varied in different groups of daylily genotypes (Table 2). The highest PIC value was observed in groups of foreign cultivars (0.215) and Lithuanian cultivars (0.202). The group of daylily species had the lowest PIC values, with an average of 0.58. The species group also had the lowest heterozygosity, while early hybrid and foreign and Lithuanian cultivar heterozygosity was at a similar level (0.68–0.71). The highest heterozygosity value was observed for the early hybrids group (1.000) with primer SAU00097. This primer was the most informative for all groups analyzed in this study according to the highest heterozygosity values (Table 2).

Molecular fingerprinting is a powerful tool for identifying and distinguishing different cultivars of plants. Phenotypic data in the AHS database and breeders’ catalogues are the only source of identifying daylily cultivars. Increasing numbers of new cultivars per year require additional tools for identifying cultivars. In breeding, microsatellite markers are commonly used to create molecular fingerprints for each cultivar. This allows breeders to track the ancestry of their plants and assess the genetic diversity within and between cultivars. Eight primer pairs were used to develop fingerprints of Lithuanian cultivars registered in the AHS (Table S2). With the same set of primers, it would be possible to determine cultivars without the need for phenotyping.

### 2.2. Genetic Relationships and Diversity of Daylily

**Figure 1:**
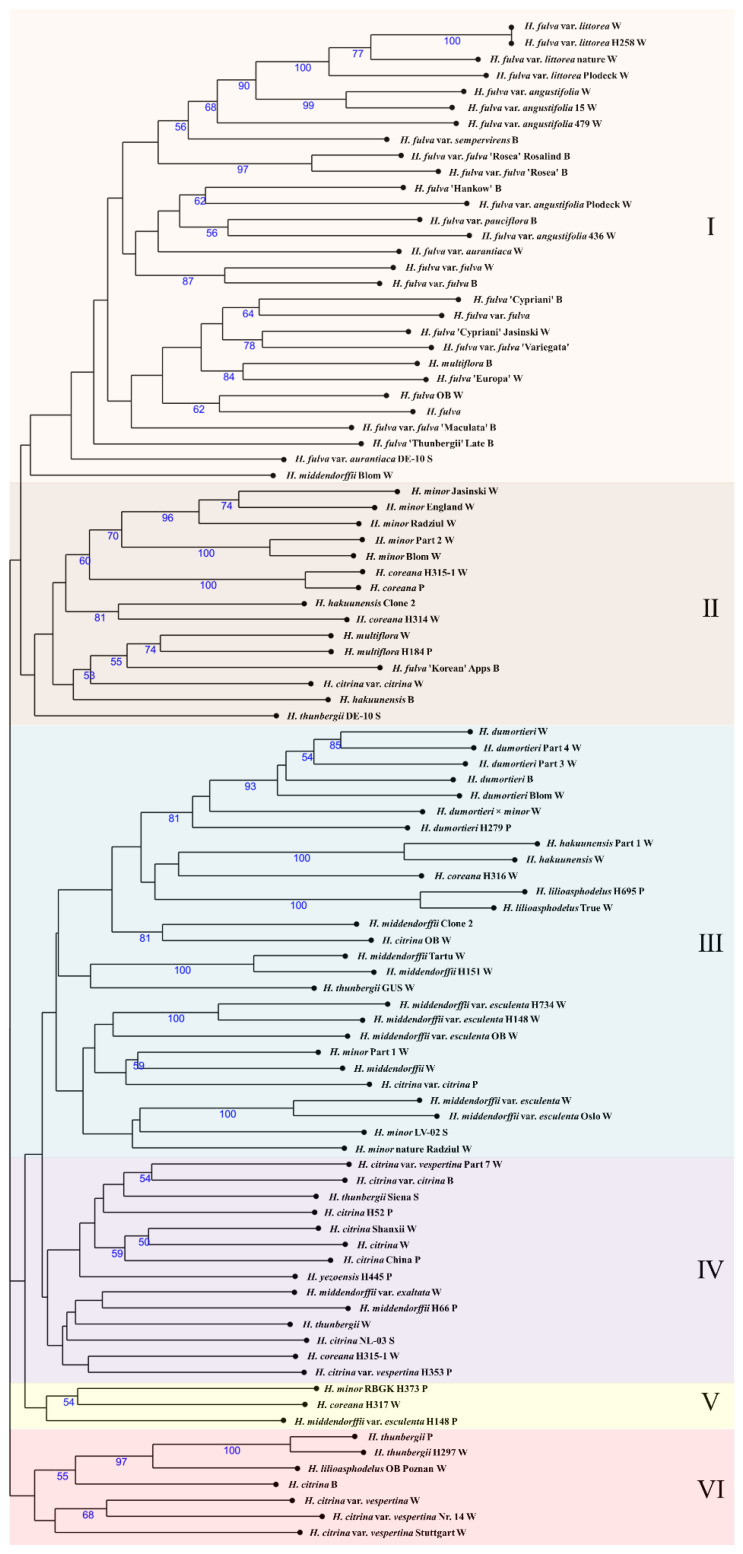
Dendrogram showing relationships among 11 species with different clones and forms of genus *Hemerocallis* based on DICE genetic distance matrix obtained by SSR. Blue numbers below branches indicate bootstrap values > 50%. The clusters of the phylogenetic tree are marked with I–VI numbers (see in the text).

In the cluster analysis, the daylily genotypes were separated into six main groups (Figure 1). The first cluster grouped mainly *H. fulva* genotypes (27), including one genotype of *H. middendorffii* and one *H. multiflora*. The second cluster contains *H. minor* (7 genotypes), *H. coreana* (3), *H. fulva* ‘Korean’ (1), *H. hakuunensis* (2), *H. multiflora* (2), *H. citrina* var. *citrina* (1), and *H. thunbergii* clone DE-10 S. The third cluster shows a branch of *H. dumortieri* (6) genotypes, along with other species *H. hakuunensis* (2), *H. coreana* (1), *H. lilioasphodelus* (2), *H. middendorffii* (4), *H. middendorffii* var. *esculenta* (5), *H. thunbergii* (1), *H. minor* (3), *H. citrina* (1), and *H. citrina* var. *citrina* (1). The fourth cluster includes *H. citrina* (5), *H. citrina* var. *citrina* (1), *H. citrina* var. *vespertina* (2), *H. thunbergii* (2), *H. yezoensis* (1), *H. middendorffii* (1), *H. middendorffii* var. *exaltata* (1), and *H. coreana* (1). The fifth cluster contains three species of *H. minor*, *H. coreana*, and *H. middendorffii* var. *esculenta*, with one genotype of each. The sixth cluster includes *H. thunbergii* (2), *H. lilioasphodelus* (1), *H. citrina* (1), and *H. citrina* var. *vespertina* (3). Only *H. fulva* and *H. dumortieri* were well-separated and grouped into clades, while other genotypes of species were dispersed in the dendrogram. In general, clusters 3 to 6 could be utilized as one with an admixture of different clones and forms of species.

**Figure 2:**
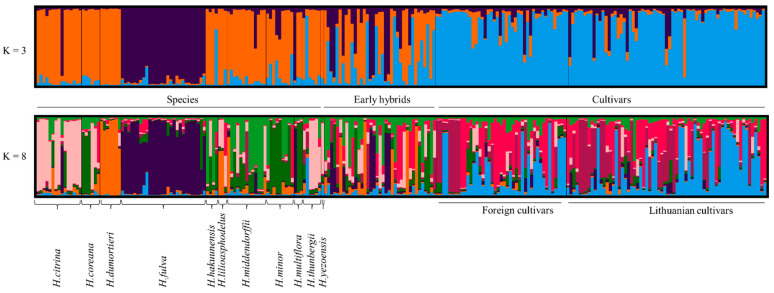
Analysis of daylily population using STRUCTURE software on 241 daylily individuals, showing genetic affinity among the studied genotypes, grouped to the optimal K = 3 by Evanno log-likelihood partitions along with K = 8 for the highest probability by K using median values of Ln (Pr data) for K. Each bar represents a different individual. In contrast, each segment’s length is proportional to the estimated membership of each group. Different colors represent main population groups.

The studied genotypes were clustered in STRUCTURE software to the two highest values of K = 3 and K = 8 (Figure 2). The most likely value of ΔK was 3 (Figure S1), indicating three genetically distinct reconstructed populations, and K = 8 (Figure S2), i.e., eight population groups. *H. fulva* was assigned as an independent group, while other species and part of the early hybrids were assigned to another group. The foreign and Lithuanian cultivars were established in a separate group. Species show an unusual pattern similar to the cultivar group. That could be because the genotype of the assigned species might originate from seeds, or the classification of species has yet to be finalized and should be revised in more complex studies. Daylily genotypes share only part of the genetic material of the parental line.

The highest source of variation was between individuals and within groups (Table 3). The highest rate of variation was observed among species scoring almost 20%. Among species, early hybrids, foreign and Lithuanian cultivars, variation was 5%, and the primary source of variation could be species and early hybrids with distinct genetic patterns (Figure 2). Variation among Lithuanian and foreign cultivars was very low since most germplasm used for Lithuanian cultivars was from abroad. This also could be because of domestication and inbreeding of similar lines.

### 2.3. Putative Origin of Early Hybrids

**Figure 3:**
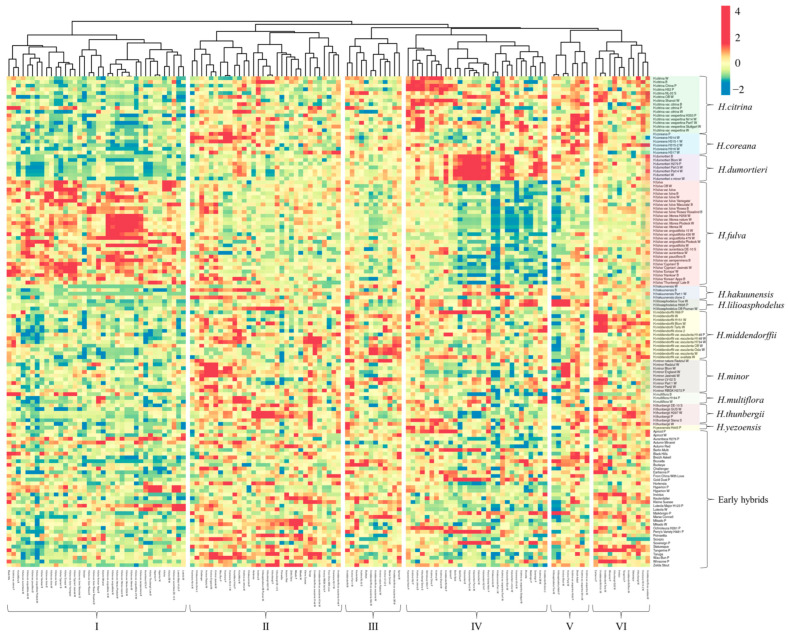
Daylily genotype similarity according to the heatmap, showing differentiation and similarity to origin species of the studied genotypes by different SSR alleles. Unit variance scaling is applied to rows. The columns are clustered (I–VI) using the correlation distance and complete linkage.

Large amounts of genetically unique genotypes within species could be traced from *H. citrina*, *H. fulva*, and *H. thunbergii* between different species found in botanical gardens. A complete linkage based on the heatmap (Figure 3) estimated the putative parents of the early hybrids. Early hybrids were analyzed and showed a complex admixture of different species involved in the breeding process. Five early hybrids have led to the distinct similarity of putative origin species: ‘Autumn Red’ and ‘Zelda Stout’—*H. thunbergii*, ‘Hortensia’—*H. coreana*, ‘Invictus’—*H. middendorffii*, and ‘Ochroleuca’—*H. citrina*.

#### 2.3.1. Diversity of Daylily Species in Early Hybrids

*H. citrina* is one of the most used species and the possible origin for several cultivars: ‘Berlin Multi’—*H. citrina* var. *citrina*, *H. dumortieri*, *H. middendorffii*, and *H. thunbergii;* ‘Breizh Askell’—*H. citrina*, *H. citrina* var. *vespertina*, *H. coreana*, *H. dumortieri*, *H. middendorffii* var. *esculenta*, *H. minor*, and *H. thunbergii;* ‘Brunette’—*H. citrina* var. *vespertina* and *H. lilioasphodelus*; ‘Buckeye’—*H. citrina* and *H. minor*; ‘Earlianna’—*H. citrina*, *H. middendorffii*, *H. minor*, and *H. thunbergii*; ‘Kleine Suesse’—*H. citrina* var. *vespertina* and *H. middendorffii* var. *esculenta*; ‘Sovereign’—*H. citrina* var. *vespertina*, *H. coreana*, *H. dumortieri*, and *H. lilioasphodelus*; ‘Taruga’—*H. citrina* var. *citrina*, *H. citrina* var. *vespertina*, *H. coreana*, *H. lilioasphodelus*, *H. middendorffii*, *H. middendorffii* var. *esculenta*, *H. minor*, *H. multiflora*, and *H. thunbergii*.

Seven early hybrids have no putative origin to the two most known species: daylily (*H. fulva*) and nightlily (*H. citrina*). These are ‘Gold Dust’—*H. dumortieri* and *H. middendorffii*; ‘Maikonigin’—*H. coreana*, *H. dumortieri*, *H. hakuunensis*, and *H. lilioasphodelus*; ‘Marse Connell’—*H. middendorffii* var. *esculenta*, *H. minor*, and *H. multiflora*; ‘Poinsettia’—*H. lilioasphodelus* and *H. thunbergii*; ‘Tangerine’—*H. middendorffii* and *H. thunbergii;* ‘Wau-Bun’—*H. coreana*, *H. hakuunensis*, *H. middendorffii* var. *esculenta*, and *H. multiflora*; ‘Winsome’—*H. thunbergii* and *H. lilioasphodelus*. These genotypes should offer new traits to breeding programs.

The hybrid of *H. dumortieri* x *minor* was similar to the genotypes of *H. dumortieri* but not to *H. minor. H. citrina* and *H. middendorffii* were the species that could be related or be the putative parent. However, cultivars ‘From China With Love’, ‘Keulenfalter’, ‘Mikado’, and ‘Scorpio’ were least similar to any species. They could be genotypes unrelated to the analyzed species.

#### 2.3.2. *H. fulva* Clones in Early Daylily Breeding Programs

*H. fulva* was observed in close similarity to ten early hybrids analyzed in this study. These include ‘Luteola Major’ H123 P—*H. fulva* var. *fulva* ‘Maculata’ and *H. fulva* var. *angustifolia*; ‘Luteola’ W—*H. fulva* ‘Thunbergii’; ‘Aurantiaca’ H276P—*H. fulva* var. *angustifolia*; ‘Autumn Minaret’—*H. fulva* var. *angustifolia*, *H. fulva* ‘Korean’, and *H. multiflora*; ‘Challenger’—*H. coreana*, *H. fulva* var. *fulva*, three hybrids of *H. fulva* including ‘Cypriani’, ‘Hankow’, and ‘Korean’, *H. middendorffii* var. *esculenta*, *H. minor*, and *H. multiflora*; ‘Statuesque’—*H. coreana*, *H. fulva*, *H. middendorffii*, and *H. thunbergii*; ‘Black Hills’—*H. citrina* var. *citrina*, *H. fulva* var. *angustifolia*, *H. fulva* Europa, *H. fulva* ‘Hankow’, and *H. multiflora*; ‘Perry’s Variety’—*H. citrina*, *H. citrina* var. *vespertina*, *H. dumortieri*, *H. fulva* var. *aurantiaca*, *H. middendorffii*, *H. middendorffii* var. *esculenta*, *H. middendorffii* var. *exaltata*, *H. minor*, and *H. multiflora*. Two clones of ‘Apricot’ from different sources share the exact putative origin of *H. citrina* and *H. middendorffi* var. *esculenta*. Nevertheless, a clone from J. Plodeck also showed *H. middendorffii* as one of a similar species. On the contrary, an Arboretum Wojsławice had two additional species: *H. fulva* ‘Thunbergii’ and *H. middendorffii* var. *esculenta*. Similarly, with ‘Hyperion’, from Arboretum Wojsławice, the putative origin species was *H. fulva* var. *angustifolia*. At the same time, a clone from J. Plodeck has the same species along with *H. fulva* var. *aurantiaca*, *H. fulva* ‘Europa’, and *H. fulva* ‘Thunbergii’. It is unknown how some of these crosses were possible, while several clones of *H. fulva* have three sets of chromosomes, i.e., they are triploid.

#### 2.3.3. Triploid *H. fulva* as Putative Origin to Both Diploid and Tetraploid Modern Daylily Cultivars

*H. fulva* var. *kwanso* (*H. fulva* var. *fulva*) is a triploid with 3n = 3x = 33 chromosomes. This specific group of species is a possible origin, along with other fulvous genotypes (Figure S3) as well as cultivars ‘Substantial Evidence’ (2x) ‘Primal Scream’ (4x—Stout medal winner), ‘Lakelet Ballerina Tutu’ (4x), ‘Shh!‘ (2x), ‘Luteola Major’ (2x), ‘Aurantiaca’, ‘Ginger Twist’ (4x), ‘Lakelet Fashion Coward’ (4x), 18–388 (2x), ‘Exotic Starfish’ (2x), ‘Mystical Elf’ (4x), ‘Go Bananas Go’ (2x), ‘Lakelet Quick Jump’ (2x), ‘Melyniu Pienas’ (2x), ‘Art Fair’ (4x), ‘Heavenly Angel Ice’ (2x and autotetraploid 4x), ‘Lakelet Another Day’ (2x), ‘Grab the Moment’ (4x), ‘One Step Forward’ (2x), ‘Black Hills’ (2x), ‘Lakelet Ready to Start’ (4x), ‘Lakelet Afterglow’ (4x), ‘Estella Cruella’ (2x), 18–498 (4x), ‘After the Fire’ (4x), ‘Webster’s Pink Wonder’ (4x or 5x), ‘Lakelet Idol Me Now’ (4x), ‘Lakelet Wild and Reckless’ (4x), ‘Lakelet Blah Blah Blah’ (4x), ‘Statuesque’ (2x), ‘Lakelet Jingle’ (2x), and ‘Voyage, Voyage’ (4x). All those cultivars are difficult maternal parents to set seedpods, especially tetraploids (4x) and pentaploid (5x) ‘Webster’s Pink Wonder’. In contrast, diploids (2x) might have gone through heterosis because they are more fertile except for ‘Substantial Evidence’ and ‘Exotic Starfish’, which were the most complicated pod parents, but pollen fertility is exceptional in some years.

## 3. Discussion

### 3.1. Population Structure, Genetic Diversity and Relationships of Daylilies

There are several ways to measure genetic diversity, but heterozygosity is one of the most used [34]. In this study, the heterozygosity was high in all four observation groups ranging from 0.583 to 0.713 (Table 2), indicating the high level of variation within a population and showing that all four groups show good breeding prospects. The other way to assess the diversity of daylilies is allelic richness. Comparing this study’s results with researchers from China [30] who analyzed mainly Chinese germplasm and wild populations, a higher allelic range with four primers (SAU00006, SAU00096, SAU00097, and SAU00150) was observed in this study (Table 1). Three primers (SAU00029, SAU00042, and SAU00176) showed a similar allelic range, while primer SAU00052 had nearly half as many alleles in this study’s genotypes than in a study from China. The more alleles there are, the higher the allelic richness, and thus the greater the genetic diversity.

For evaluation of variation among and within groups of daylilies, the AMOVA analysis was performed. This provided the insight that the highest variation among genotype groups was observed in the species, while modern cultivars had the slightest variation among genotypes. However, the genetic variation among species was reasonably low (18%, Table 3) and indicated possible inbreeding. On the other hand, since the primary pool of daylilies for selection was only several selected genotypes from the wild population [12] and the natural feature of daylilies is self-incompatible [9,10], it could also influence the poor genetic diversity of species found in breeding programs [35]. This indicates the need to introduce more wild genotypes into breeding programs of daylilies to extend the gene pool. A similar problem is seen in other plants: *Nebulo* [36], *Oryza* [37], *Lycoris* [38], *Rhododendron* [39], and *Narcissus* [40] species to overcome the bottleneck of low genetic diversity and gene flow. Increasing genetic variation in daylily breeding programs by adding modern cultivars and early hybrids and species could ensure the diversity of new cultivars.

The fingerprinting of daylily genotypes is an essential tool for tracking true-to-name genotypes. RAPD, ISSR, and SSR unique markers could be generated and utilized for clonal fidelity analysis on specific cultivars [41]. Since there are circulating daylilies with the same name, they show different fingerprints. Wild populations of daylilies growing across China are relatively genetically diverse [30]. However, different populations of daylilies had varying degrees of genetic similarity. This suggests that there has been some mixing between populations over time. However, there could be another issue with early hybrids, where hybridizers released seedlings of the same combination under one name. These cultivars share similar fingerprints but are not identical. Current hybridizers and growers understand that daylilies multiply only by division and that an individual seedling differs from its sibling by at least one feature and distributes only selected and vegetatively propagated clones.

### 3.2. Daylily Species Classification and Implication for Modern Breeding Programs

In our study, *H. aurantiaca* was classified as an early hybrid by Dr. Jürg Plodeck along with *H. fulva* var. *aurantiaca* from Arboretum Wojsławice Botanical Garden and Šiauliai botanical garden. Two genotypes were similar to each other as well as to *H. fulva* var. *littorea*, *H. fulva* var. *angustifolia*, and even the *H. middendorffi* H66 clone according to the heatmap (Figure 3). In contrast, the Jürg genotype did not have genetic similarity to *H. fulva* var. *aurantiaca* clones and was more similar to *H. fulva* var. *fulva* and *H. fulva* var. *angustifolia*. It shows possible inbreeding with other species of the genus and raises awareness of classification issues within species, forms, and cultivars known to breeders. Amplified fragment length polymorphism (AFLP)-based QTL mapping studies in *H. fulva* and *H. citrina* wild populations showed that *H. fulva* heterozygosity was higher than *H. citrina* [42]. Upadhyay et al. [43] suggested the use of AFLP in clonal and synonym genotypes, since SSR markers did not generate unique fingerprints that would enable more reliable identification. Random amplified polymorphic DNA (RAPD) was previously used to estimate the genetic variation and relationships among species and varieties in *Hemerocallis* native to Japan, showing that *H. aurantiaca* and *H. fulva* were genetically closely related [44]. Study of grape accessions using both microsatellite and RAPD analysis showed that both methods could be utilized to analyze genetic relationships, but microsatellite primers were more informative than RAPD [45].

Using SSR molecular markers, the *H. fulva* group was distinctly separated from other species. The same tendency has been observed using AFLP analysis [11]. The middendorffii group, according to Erhardt [46], consists of *H. dumortieri*, *H. middendorffii*, and *H. hakuunensis* species. Using SSR markers, *H. lilioasphodelus* was additionally assigned to this group, which was also seen in the previous Tomkins study [11]. However, the distinction between the citrina group and middendorffii was not defined and contained an overlap of *H. citrina* and *H. minor*. The citrina group should consist of *H. lilioasphodelus* and *H. thunbergii*. Nevertheless, Tomkins’s study suggests that the middendorffi and citrina groups should be merged into one large taxonomic group [11]. This study can confirm that fulvous daylilies are separated, and the rest of the species were distributed into different groups but not separated into individual groups, as Erhardt [46] suggested. The Blom *H. middendorffii* clone from Wojsławice Botanical Garden was grouped in the same clade with fulvous genotypes (Figure 1). The same genetic relationships of *H. middendorffii*, *H. fulva*, and *H. fulva* var. *fulva* (in article *H. fulva* f. *Kwanso*) were observed in a phylogenetic study in Korea [47], indicating that *H. middendorffii* could be the origin of fulvous daylilies. Other clones of this species were clustered into other clades. In this study, 3–28 individuals represent a species or species form. Therefore, there is not a sufficient number of genotypes for all self-incompatible species to represent the population’s genetic diversity. This may be another reason that individual species’ genotypes fell into separate clusters in the phylogenetic tree (Figure 1). Resolving this question would require detailed systematic and genetic studies of more different accessions of species at their sites of origin.

The main confusion is with *H. citrina*, where different clones from different sources are not forming species clusters (Figure 1). The complex of *H. middendorffii* species in Japan highly reflects the geographical features [48], but it denies taxonomy, pointing out that intraspecific clones have recent taxonomic entities and lineage admixture generating novel phenotypic populations with complex ancestry [28,29]. *H. middendorffii* was widely distributed in this study, but var. *esculenta* was grouped in one branch (Figure 1). Hirota et al. [49] observed an inconsistency of genome phylogeny using SNP for cpDNA, pointing out that interspecific gene flow corresponds to floral traits and is weaker than the effect of floral traits alone [49]. That could be caused by flowering time and duration.

Daylily species are classified as day and night lilies. It is known that *H. fulva* is considered a daylily (open between 3:00 and 15:00), while *H. citrina* and *H. lilioasphodelus* are nightlilies (open between 15:00 and 3:00 of the next day) [1]. In previous studies, daylilies and nightlilies were grouped into different groups [30], and some accessions had an admixture of the two groups. *H. fulva* also formed a different group from nightlilies (Figure 1). This unique feature in one genus of plants limits the natural mating of different species in the wild. However, for daylily breeders, this could open new features of crossing both types to create extended blooming time daylilies. However, F1 from wild species did not show a blooming time of more than 12 h [50,51], while hybrids from cultivars bloomed for more than 12 hours [1]. The current daylily rust outbreak in Europe raises awareness of resistance genotypes. Ramos et al. [52] state that European daylily cultivars ‘Cherry Tiger’, ‘German Highlight’, and ‘Romantic Rose’ are moderately resistant, while others are susceptible to rust. There are no data on daylily species’ resistance to rust. Daylily breeders should consider introducing different genotypes of daylily species into breeding lines to enrich the genetic pool and reintroduce resistance to biotic and abiotic stresses.

### 3.3. Ploidy in Breeding Programs

Examination of daylily ploidy showed the occurrence of triploids or pentaploids [53]. Such polyploids are induced by antimitotic agents [53] or by misinterpreting ploidy levels during registration [54]. This shows uncommon phenomena in plants of triploid existence and even the possibility of mating. In wild types examined in China, 45% were triploids, while the rest were diploids without natural tetraploids [55]. Triploid daylilies show greater leaf length, plant height, and broader leaves than diploids and tetraploids [54]. It is possible to cross diploids with tetraploid daylilies and triploids with tetraploids, with a very low possibility of crossing diploids with triploids [56]. There are indications that some early hybrids and cultivars evolved from triploid daylilies since they were grouped in the same group (Figure 1). Hybridizers using these cultivars in their breeding works could face fertility problems. However, it also indicates why induced daylily polyploids had uneven ploidy levels, atypical of somatic ploidy induction using antimitotic agents in other plants. Nevertheless, it should be analyzed and tested for further clarification.

## 4. Materials and Methods

Plant material and DNA extraction. Plant material for this study was collected in 5 collections: Bourdillon (France), Jürg Plodeck (Germany), Vilnius University Šiauliai Academy (VU ŠA) Botanical Garden (Lithuania), University of Wrocław Botanical Garden Arboretum Wojsławice (Poland), and Edvinas Misiukevičius (Lithuania). In total, 241 genotypes were used in this study. They were grouped by origin into four groups: species, early hybrids, foreign cultivars, and Lithuanian cultivars. A total of 65 Lithuanian genotypes, including 57 cultivars, were created in Lithuania, and there were 44 foreign cultivars, 37 early hybrids, and 95 genotypes from 11 species groups, including forms known to botanists. All plants were grown in one location, and young leaves were collected and flash-frozen for DNA extraction. The modified Cetyl Trimethyl Ammonium Bromide (CTAB) method [57] was used for DNA extraction. DNA was dissolved in 100 µL of TE buffer. The extracted DNA was quantified using a NanoDrop spectrophotometer normalized to a concentration of 200 ng/µL and kept at −70 °C until further analysis.

SSR Analysis. Polymerase chain reaction (PCR) using ten previously published SSR primers [30] (Table S3) was performed with Eppendorf Mastercycler x50a (Eppendorf, Germany). PCR amplifications were performed with 10 µL total volume of the reaction mixture, consisting of (300 ng/µL) DNA, 0.2 mM of each primer, 25 mM of MgCl, 2 mM dNTP, 10 × buffer, 10 mM DTT, 1% PVP, 500 U Taq DNA polymerase (Thermo Scientific, USA). The PCR was performed with the conditions as follows: initial denaturation at 94° for 10 min followed by five cycles at 94° for 30 s, 65° for 45 s and 72° for 1 min with touchdown procedure at primer annealing step (−1 °C in each cycle), 30 cycles 64° for 45 s, 60° for 45 s and 72° for 1 min, with a final extension step of 10 min at 72°. The agarose gel was used for pre-screening the SSR primers. Eight out of ten used SSR primer pairs showed amplicons, and the forward primer was labelled with a fluorescence dye (Table S3). Capillary electrophoresis was performed with Genetic Analyser 3130 (Applied Biosystems, Foster City, CA, USA).

Data Analysis. The informativeness of the SSR primer was investigated according to the number of alleles, observed heterozygosity (H_0_), and the polymorphism information content (PIC). PIC values were calculated for each pair of SSR primers according to Roldán-Ruiz et al. [58].

The phylogenic dendrogram of *Hemerocallis* species, clones, and forms was constructed based on the DICE similarity distance matrix generated using XLSTAT [59] and UPGMA methods within DARwin 6.0.021 programme [60] with 30,000 bootstraps. To investigate the population structure, Structure software [61] was used to determine the groups of populations in this study. The Bayesian cluster analysis was performed with STRUCTURE software based on 8 SSR primers for all 241 genotypes. The analysis was performed with 10,000 iterations each plus 10,000 iterations as burn-in. The analysis was run starting with K = 1 and finishing at K = 30. All Structure analyses with 20 independent runs for each K value were summarized on the online platform Harvester [62], which uses the Evanno method [63] to assess the most likely K value given the data. Produced combined files from 20 replicates for the best K were run and used in Clumpp [64] to identify clustering modes and to package population structure inferences across K. To test whether species, early hybrids, foreign and Lithuanian cultivars are biologically meaningful, Analyses of Molecular Variance (AMOVA) were performed using GenAlEx software [65,66], analyzing among and within groups as well as individual differentiation with 10,000 permutations in each. The phylogenetic tree could show only the possible origin of just one parental line. To identify the possible origin of early hybrids, a heatmap was used and constructed using the ClustVis Web tool [67] to visualize the clustering of multivariate data based on the DICE similarity distance matrix.

## 5. Conclusions

In this work, we have characterized the genetic diversity of early hybrids and cultivars using microsatellite simple sequence repeat (SSR) markers and attempted to identify the origin of *Hemerocallis* spp. early hybrids. Molecular fingerprints of daylilies were developed using eight primer pairs. The highest number of alleles was generated with primers SAU00042, SAU00096, and SAU00097. Primer SAU00097 has the highest heterozygosity and PIC value, making this primer valuable in molecular studies of different daylily genotypes. The highest heterozygosity was observed in Lithuanian cultivars (0.713) and the lowest in species (0.583). Genetic relationships between species show only fulvous daylilies being separated into the different clades. The highest variation among genotypes was observed in the species group, while the modern cultivars had the least variation among genotypes. The putative origin of early hybrids was assigned from the likelihood heatmap of all genotypes, which shows the species used in breeding early hybrids. This provides insights into the breeding programs of daylilies and allows hybridizers to track the ancestry of their plants and assess the genetic diversity by using a broader range of genotypes, including not only modern daylilies but also wild species. The nomenclature of daylily species remains complicated. More research is required to understand the relationships between different species, their representative genotypes, and new groups of modern cultivars. Unravelling why triploid daylilies occurred in the wild and how that influenced the ploidy of daylily germplasm for centuries would also be interesting.

## Figures and Tables

**Table 1:** SSR primer informativeness for daylily germplasm collection, including species, early hybrids, and modern cultivars.

Locus	No. of Alleles	Observed Size Range, bp	H_0_^a^	PIC ^b^
SAU00006	14	225–255	0.53	0.17
SAU00029	16	225–266	0.43	0.14
SAU00042	24	182–219	0.67	0.13
SAU00052	11	93–118	0.66	0.16
SAU00096	25	161–223	0.78	0.13
SAU00097	26	94–130	0.95	0.17
SAU00150	19	272–299	0.69	0.14
SAU00176	20	215–268	0.56	0.13
SAU00008	n.a. ^c^	n.a.	n.a.	n.a.
SAU00048	n.a.	n.a.	n.a.	n.a.
Mean	19	_	0.66	0.15

^a^ H_0_—observed heterozygosity, ^b^ PIC—polymorphism information content, ^c^ n.a.—not available.

**Table 2:** SSR primer informativeness in 4 groups representing evolutionary distinction of daylily.

Locus	H_0_	PIC	H_0_	PIC	H_0_	PIC	H_0_	PIC
	Species		Early hybrids		Foreign cultivars		Lithuanian cultivars	
Investigated genotypes	95		37		44		65	
SAU00006	0.495	0.225	0.703	0.227	0.523	0.238	0.439	0.221
SAU00029	0.453	0.175	0.487	0.252	0.341	0.212	0.386	0.167
SAU00042	0.463	0.114	0.838	0.170	0.796	0.214	0.807	0.224
SAU00052	0.568	0.167	0.757	0.241	0.727	0.232	0.649	0.198
SAU00096	0.779	0.144	0.649	0.175	0.796	0.189	0.825	0.186
SAU00097	0.916	0.163	1.000	0.187	0.977	0.217	0.947	0.211
SAU00150	0.537	0.135	0.730	0.163	0.750	0.201	0.842	0.188
SAU00176	0.453	0.146	0.432	0.177	0.523	0.218	0.807	0.222
Mean	0.583	0.159	0.700	0.199	0.679	0.215	0.713	0.202

**Table 3:** Analysis of molecular variance (AMOVA) on microsatellite data across the daylily species and cultivar groups.

Source of Variation	df	SS	MS	Est. Var.	%
10 species: *H. citrina*, *H. coreana*, *H. dumortieri*, *H. fulva*, *H. hakuunensis*, *H. lilioasphodelus*, *H. middendorffii*, *H. minor*, *H. multiflora* and *H. thunbergii*					
Among species	9	228.245	25.361	1.885	18%
Within populations and species	84	741.372	8.826	8.826	82%
Among individuals	93	969.617		10.711	100%
Four groups, combining species, early hybrids, foreign and Lithuanian cultivars					
Among groups	3	125.613	41.871	0.540	5%
Within groups	237	2571.764	10.851	10.851	95%
Among individuals	240	2697.378		11.391	100%
Two groups, combining cultivars of Lithuanian and foreign cultivars					
Among groups	1	16.722	16.722	0.105	1%
Within groups	107	1198.104	11.197	11.197	99%
Among individuals	108	1214.826		11.303	100%

Note: SS—sum of the square; MS—mean square; Est. Var.—estimated variance; %—percentage of variance.

## Data Availability

Not applicable.

## Supplementary Materials

The following supporting information can be downloaded at: https://www.mdpi.com/article/10.3390/plants12091752/s1; Figure S1: DeltaK graph with optimal K by Evanno; Figure S2. Probability by K graph using medial values of Ln(Pr Data) the K; Figure S3: Daylily genotype similarity clustering using heatmap. Table S1: The sampling location and ploidy level of daylily genotypes; Table S2: Molecular fingerprints of daylily cultivars developed in Lithuania using SSR; Table S3 Li et al. [30] SSR primers used for the evaluation of daylily genetic diversity.
