# Supplementary figures and images for: Assessing the Genetic Diversity of Daylily Germplasm Using SSR Markers: Implications for Daylily Breeding
Source: Plants (Basel). 2023 Apr 25;12(9):1752. doi: 10.3390/plants12091752 (PMC10181390; doi:10.3390/plants12091752)

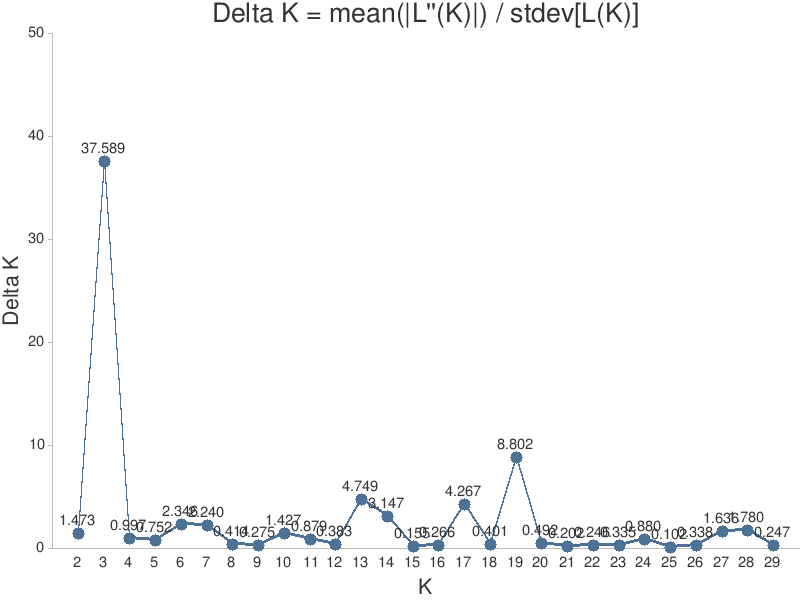

Supplement: Supplementary file 1 [file plants-12-01752-s001.zip › Figure S1. DeltaK graph with optimal K by Evanno..png]

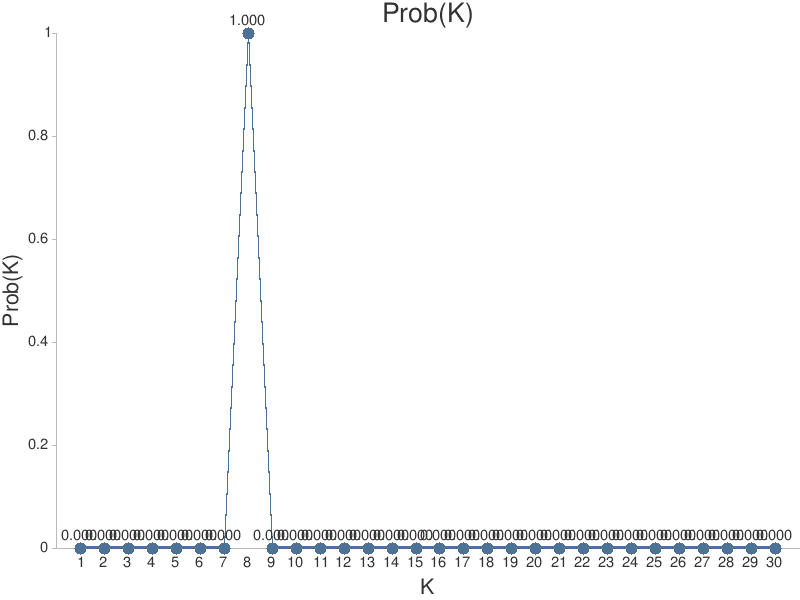

Supplement: Supplementary file 1 [file plants-12-01752-s001.zip › Figure S2. Probability by K graph using medial values of Ln(Pr Data) the K..png]

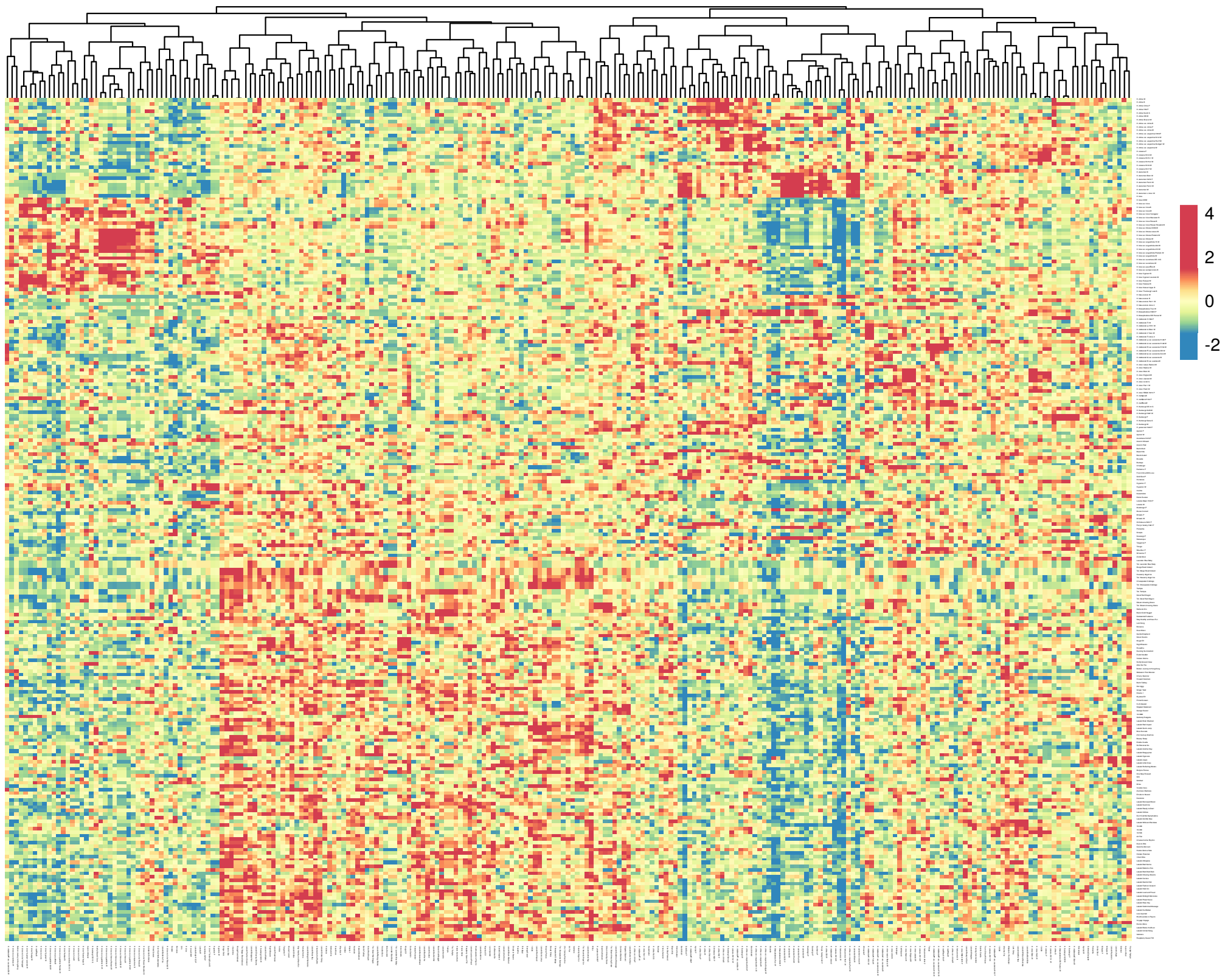

Figure S3. Daylily genotype similarity clustering using heatmap

Supplement: Supplementary file 1 [file plants-12-01752-s001.zip › Figure S3. Daylily genotype similarity clustering using heatmap.pdf]
